# An overview of geospatial methods used in unintentional injury epidemiology

**DOI:** 10.1186/s40621-016-0097-0

**Published:** 2016-12-26

**Authors:** Himalaya Singh, Lauren V. Fortington, Helen Thompson, Caroline F. Finch

**Affiliations:** 1 Australian Collaboration for Research into Injury in Sport and its Prevention (ACRISP), Federation University Australia, SMB Campus, PO Box 663, Ballarat, 3353 Australia; 2 School of Health Sciences and Psychology, Faculty of Health, Federation University Australia, Ballarat, Australia; 3 Centre for eResearch and Digital Innovation (CeRDI), Federation University Australia, Ballarat, Australia

**Keywords:** Geographical epidemiology, Spatial epidemiology, Mapping, Spatial analysis, Smoothing, Clustering, Cluster detection, Geographical correlation, Ecological analysis

## Abstract

**Background:**

Injuries are a leading cause of death and disability around the world. Injury incidence is often associated with socio-economic and physical environmental factors. The application of geospatial methods has been recognised as important to gain greater understanding of the complex nature of injury and the associated diverse range of geographically-diverse risk factors. Therefore, the aim of this paper is to provide an overview of geospatial methods applied in unintentional injury epidemiological studies.

**Methods:**

Nine electronic databases were searched for papers published in 2000–2015, inclusive. Included were papers reporting unintentional injuries using geospatial methods for one or more categories of spatial epidemiological methods (mapping; clustering/cluster detection; and ecological analysis). Results describe the included injury cause categories, types of data and details relating to the applied geospatial methods.

**Results:**

From over 6,000 articles, 67 studies met all inclusion criteria. The major categories of injury data reported with geospatial methods were road traffic (*n =* 36), falls (*n =* 11), burns (*n =* 9), drowning (*n =* 4), and others (*n =* 7). Grouped by categories, mapping was the most frequently used method, with 62 (93%) studies applying this approach independently or in conjunction with other geospatial methods. Clustering/cluster detection methods were less common, applied in 27 (40%) studies. Three studies (4%) applied spatial regression methods (one study using a conditional autoregressive model and two studies using geographically weighted regression) to examine the relationship between injury incidence (drowning, road deaths) with aggregated data in relation to explanatory factors (socio-economic and environmental).

**Conclusion:**

The number of studies using geospatial methods to investigate unintentional injuries has increased over recent years. While the majority of studies have focused on road traffic injuries, other injury cause categories, particularly falls and burns, have also demonstrated the application of these methods. Geospatial investigations of injury have largely been limited to mapping of data to visualise spatial structures. Use of more sophisticated approaches will help to understand a broader range of spatial risk factors, which remain under-explored when using traditional epidemiological approaches.

**Electronic supplementary material:**

The online version of this article (doi:10.1186/s40621-016-0097-0) contains supplementary material, which is available to authorized users.

## Review

### Background

Injury is a leading preventable cause of death and disability around the world (Peden et al. 2002). Previous epidemiological studies have demonstrated that injury incidence is often related to external socio-economic and physical environmental factors (Muller et al. 2005; Poulos et al. 2007). Unlike many non-communicable health-related conditions, the incidence of many injuries can also be directly linked to specific places (e.g. body of water, road intersection, junctions) (Dai et al. 2013; Lai et al. 2011; Zhang et al. 2015). Therefore, to better understand injury causation, it is important to account for the interplay between social and environmental risk factors in relation to their geographic (or spatial) distribution (Bell and Schuurman 2010). Geographic Information System (GIS) tools and geospatial analysis methods can be used to investigate these spatial risk factors, which have been under-explored in traditional epidemiological studies (Beale et al. 2008; Ostfeld et al. 2005).

Geospatial methods have a long history of use in public health, including for epidemiological research (Auchincloss et al. 2012; Lawson 2001). Within this area, termed spatial epidemiology, investigations can be characterised by three broad categories of enquiry: (i) mapping; (ii) clustering/cluster detection (hotspot analysis); and, (iii) ecological analysis (Elliott and Wartenberg 2004; Lawson 2001; Lawson et al. 2016; Singh et al. 2015). These categories are interrelated, and may overlap in some cases, so they should not be considered as distinct components (Elliot et al. 2000; Lawson et al. 2016).

### Category 1: mapping

Mapping has primarily been used to describe disease incidence in a spatial context and subsequently, to formulate aetiological hypotheses by identifying areas of high-risk (Elliot et al. 2000; Lawson et al. 2000). The choice of map depends largely on the spatial resolution of the available data. In public health, this data tends to be based on specific point features (e.g. residential addresses or coordinates of disease location) or aggregated by areal features (e.g. state, county, local government area or postcode.) To represent point data, a point map is commonly used, in which each individual case is represented by a single point on a map relative to its geographic location (Waller and Gotway 2004). This is useful when study aims include understanding how individual cases are distributed across space. To represent attribute information associated with individual cases, other types of point maps can be used such as graduated colour maps where a range of colours (e.g. blue to red) indicate a progression of numeric values. Where areal data is available, the choropleth map is commonly used, in which different colour patterns are applied to regions representing a class of values (Waller and Gotway 2004). Other types of maps, such as classed symbol maps, are less commonly used.

Most commonly, disease data is available as aggregated summaries for areal features such as postcode, census tract or counties (Beale et al. 2008). Statistical techniques are then applied to estimate area level risks, and those estimates are mapped to understand the spatial distribution of risk. The most common summary measures of occurrence are frequency, incidence rates, standardised mortality ratio and relative risk (Beale et al. 2008). When counts or rates are large, their distributions follow statistical assumptions inherent in linear models. However, if the counts or rates are small, as is the case in some areas, the application of appropriate smoothing techniques are required to address the small number problem (Waller and Gotway 2004).

### Category 2: clustering/cluster detection

Clustering/cluster detection refers to the uncovering of “unusual” aggregation of disease incidence (Fritz et al. 2013; Lawson 2001). These methods are applied to investigate how health outcome data relate spatially by identifying: (i) the presence of any clusters, in which case global (general, non-specific) methods are used; and, (ii) the location of clusters in space, for which local (focused, specific) methods are used (Lawson 2001; Lawson et al. 2016). Usually, global methods generate an autocorrelation parameter that defines the nature of the spatial pattern whereas local methods identify the specific locations of clusters, also known as hotspots. Many clustering/cluster detection methods have been developed based on different statistical models specific for point and/or areal features within the two broad categories of global and local (Fritz et al. 2013). Such methods are underpinned by different statistical approaches, so each method could provide different clustering/cluster results for the same set of data (Waller and Gotway 2004).

A review that summarised the clustering/cluster detection methods most commonly applied in epidemiology identified Diggle and Chetwynd’s bivariate K-function, Mantel-Bailar’s test and the Potthoff-Whittinghill method as the most preferred global methods and spatial scan statistics as the most preferred local method (Auchincloss et al. 2012). A more recent summary reviewed cluster methods applied in epidemiology for point data and identified that the K-function is the most commonly used global method followed by methods based on the nearest neighbour statistics such as nearest neighbour index (NNI), nearest neighbour hierarchical (NnH) and Cuzick Edwards test (Fritz et al. 2013). The study also reported the most common local method to be spatial scan statistics (Fritz et al. 2013). Other methods have also been used in broader public health applications such as kernel density estimation, Moran’s I, Local Indicator of Spatial Autocorrelation (LISA), Getis Ord statistics, and Tango’s maximized excess events test (Auchincloss et al. 2012; Fritz et al. 2013). Each clustering/cluster detection method has its own strengths and weaknesses and may not be appropriate to all datasets because each dataset differs in spatial resolution (point or areal), spatial coverage (area covered by dataset) and spatial intensity (distribution of outcome of interest) (Fritz et al. 2013; Waller and Gotway 2004).

### Category 3: ecological analysis

Ecological analyses examine the spatial distribution of disease incidence in relation to explanatory factors (Lawson et al. 2016). These types of studies use spatial statistical models to investigate the relationship between exposures and disease at an aggregate level (Elliot et al. 2000; Lawson et al. 2016). Importantly, traditional statistical models may not be appropriate for the analysis of spatially dependent data because of their inability to address or account for spatial autocorrelation and/or spatial heterogeneity. Spatial regression models have therefore been developed under both frequentist and Bayesian approaches, with common methods used in epidemiological studies being Conditional Autoregressive Models (CAR), Geographically Weighted Regression (GWR) and the Besag York and Molliè (BYM) approach (Auchincloss et al. 2012; Chaney and Rojas-Guyler 2016; Rezaeian et al. 2007). These methods differ in their complexity of computation, approach towards capturing spatial heterogeneity, and in how they quantify the uncertainty associated with parameter estimates (Auchincloss et al. 2012).

### Aim of the review

While the principles of geospatial analysis have broad relevance to injury epidemiology, their application to injury data is still relatively novel (Bell and Schuurman 2010; Cusimano et al. 2007; Singh et al. 2015). One possible reason for this could be that geospatial analysis requires spatially referenced health and determinant data at a population level (Beale et al. 2008; Bell and Schuurman 2010). With widespread use of global positioning system (or GPS) technologies over the past decade, these data have become increasingly available and can now be linked to injury data sets. In addition, wider accessibility to GIS for the management, analysis and presentation of spatial data has also increased in the last decade, with capability now (at least partially) incorporated into standard statistical software (e.g. STATA (StataCorp 2015)) or available through open source platforms (e.g. QGIS (QGIS 2015), GeoDa (Anselin et al. 2006), SatScan (Kulldorff et al. 1998), CrimeStat (Levine 2000)). Given the increase in availability of both spatially-referenced injury data and GIS software, it is timely to consider how and when geospatial methods have been applied to injury epidemiology studies.

A previous review summarised the history of GIS in relation to injury prevention (Bell and Schuurman 2010), but that review did not include details about the actual geospatial methods used in the published literature. Therefore, the aim of this study is to summarise the application of geospatial methods to unintentional injury as found in epidemiological studies published since 2000. The focus is on the type of analysis and/or data representation approach used, rather than on the injury incidence estimates per se. The intention is for these new review findings to help inform future research agendas in injury prevention.

## Methods

The publication search was guided by the Preferred Reporting Items for Systematic Reviews and Meta-Analyses (PRISMA) guidelines (Additional file 1) (Moher et al. 2009). As the aim was to summarise the geospatial analysis methods reported in each study, some items of the PRISMA statement were not applicable (e.g. there was no formal assessment of risk of bias), nor was a quality assessment of the reviewed studies undertaken given the focus was on the adopted analysis methods only.

### Search strategy

The focus of the review was restricted to unintentional injury studies given the strong link between the occurrence of such events and a specific single geographic location (e.g. a road intersection, body of water). A comprehensive list of MeSH terms and free text keywords relating to geospatial methods and unintentional injury incidence were used to develop a search strategy (Additional file 2). Nine electronic databases were searched: Medline, Academic Search Complete, CINAHL Complete, Engineering Source, GeoRef, Health Source: Nursing/Academic Edition, PsycINFO, SPORT Discus with Full Text, Web of Science.

### Study selection and eligibility

Standardised inclusion and exclusion criteria were formulated (Additional file 2) and independently applied by two authors to scan the title and abstract of all search results. Any publication deemed potentially eligible was included for full text review.

Full text review determined if studies investigated unintentional injuries using geospatial methods to address one or more of the following aims:

- To describe the geographical/spatial variation of injury incidence;
- To test for clustering or to identify clusters;
- To address aetiological questions (provide aetiologic cues about the relationship between the spatial distribution of injury incidence and explanatory factors at the aggregate level).

There was a large number of studies initially included that were subsequently identified as not reporting injury data. In particular, there were a large number of road transport studies that reported data in terms of crashes, collisions or accidents rather than reporting the frequency or rate of the injuries sustained during such events (Blazquez and Celis 2013; Zhang et al. 2015). Only studies where injuries were clearly identifiable were retained (as opposed to those with a focus on potential injury-causing events). Original peer-review studies, published in 2000 to 2015, were included.

Studies that investigated intentional injuries, such as suicides or violence, were not included. We have excluded studies focused on assessing spatial access to trauma centres because our aim is to summarise methods used for epidemiological investigation rather than those associated with healthcare resource planning.

### Data extraction

Descriptive data from each study was extracted by the first author (Additional file 3). Where information was unclear or inconsistent, it was discussed with co-authors until agreement was reached on an outcome. The extracted data and definition of terms sought from each study were:

- First author and year of publication: to identify specific studies and to assess the use of geospatial methods over time.
- Injury causes: to categorise each study as being focussed on one or more of the following external cause categories—road traffic, falls, drowning, burns, poisoning, natural disasters, and others (including combined causes).
- Data coverage: to identify the source of the data and its geographic location.
- Name of the GIS package used to analyse the spatial data.
- Study classification: Studies were classified into one or more of the three broad categories of spatial epidemiological approaches, and relevant details of the methods applied in each category were extracted.
- Mapping studies: To be classified in this category, studies had to report one or more maps representing raw injury data or results derived from statistical models applied to that injury data for descriptive purposes. The information extracted from each paper included data relevant to the type of map (e.g. point, choropleth, classed symbol), the summary measure considered (e.g. incidence rates, standardised mortality ratio) and any smoothing technique (e.g. empirical Bayes method, BYM) applied.
- Clustering/cluster detection studies: To be included in this category, studies had to apply one or more methods to the injury data to test for clustering (as a measure of spatial autocorrelation or spatial heterogeneity or spatial dependency) or to identify clusters (also known as hotspots). Information regarding each method in terms of its spatial resolution (point or areal), and approach (global or local) were extracted.
- Ecological studies: To be classified in this category, studies had to apply one or more spatial regression methods to address aetiological questions with the question clearly stated in the study objective. The applied method, as well as the dependent and type of explanatory variables used in the analysis, were extracted.

### Analysis of extracted data

Studies were grouped by injury cause categories, publication year and geospatial analysis approach/es. Summaries of the extracted data were tabulated and summarised in text.

## Results

**Fig. 1:**
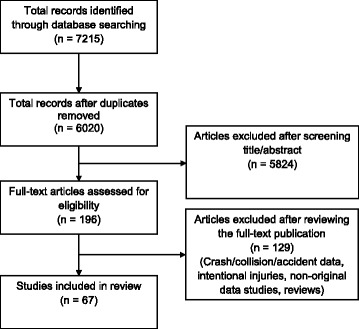
Flowchart of selection process for studies that applied geospatial methods to investigate unintentional injuries

From more than 6,000 publications identified, 67 studies met all criteria for inclusion (Fig. 1).

The majority of studies were concerned with road traffic injuries (*n =* 36) (Chakravarthy et al. 2010; Cinnamon et al. 2011; DiMaggio 2015; Dissanayake et al. 2009; Durkin et al. 2005; Eksler and Lassarre 2008; Eksler et al. 2008; Erdogan 2009; Haynes et al. 2005; Haynes et al. 2008; Hijar et al. 2003; Hosking et al. 2013; Hu et al. 2008; Huff et al. 2012; Jones et al. 2008; La Torre et al. 2007; Lassarre and Thomas 2005; Lateef 2011; Lawrence et al. 2015; Mohan et al. 2015; Morency and Cloutier 2006; Nagata et al. 2011; Nunes and Nascimento 2012; Nunn and Newby 2015; Paulozzi 2006; Poulos et al. 2012; Razzak et al. 2011; Schuurman et al. 2009; Silva et al. 2011; Slaughter et al. 2014; Spoerri et al. 2011; Statter et al. 2011; Sukhai et al. 2009; Unni et al. 2012; Weiner and Tepas 2009; Yan-Hong et al. 2006). Other studies considered falls (*n =* 11) (Bamzar and Ceccato 2015; Chan et al. 2012; de Pina et al. 2008; Dey et al. 2010; Lai et al. 2009a; Lai et al. 2009b; Lai et al. 2011; Morency et al. 2012; Towne et al. 2015; Turner et al. 2009; Yiannakoulias et al. 2003), burns (*n =* 9) (Edelman et al. 2010; Fouillet et al. 2006; Goltsman et al. 2014; Harlan et al. 2013; Heng et al. 2015; Mian et al. 2014; Niekerk et al. 2006; Stylianou et al. 2015; Williams et al. 2003), drowning (*n =* 4) (Dai et al. 2013; Maples and Tiefenbacher 2009; Sharif et al. 2012; Shenoi et al. 2015), occupational (*n =* 2) (Breslin et al. 2007; Forst et al. 2015), aviation-related (*n =* 2) (Grabowski et al. 2002a, 2002b), poisoning (*n =* 1) (Nkhoma et al. 2004), natural disaster (*n =* 1) (Peek-Asa et al. 2000) and dog-bite (*n =* 1) (Raghavan et al. 2014).

### Adopted geospatial analysis approaches

Mapping was the most common approach applied to the geospatial data, being reported in 93% (*n =* 62) of the included publications. Clustering or clustering detection methods were used in 40% (*n =* 27) and spatial regression methods for ecological analysis were applied in only 4% (*n =* 3) of studies. As Table 1 shows, some studies used >1 approach, so the percentage of studies using each approach does not sum to 100%. The majority of studies (*n =* 46, 67%) reported only one analysis approach, most commonly mapping, but 18 (27%) used two approaches and three (4%) studies reported all approaches.

**Table 1:** Number of studies (*n =* 67) across the three categories: mapping, clustering/cluster detection and ecological analysis

	Spatial epidemiological approach categories			Total studies
	Mapping	Clustering/cluster detection	Ecological analysis	
Mapping only	√	-	-	41
Cluster only	-	√	-	5
Mapping/cluster	√	√	-	18
All categories	√	√	√	3
				67^a^
Total approaches^a^	62	27	3	92^a^

^a^The total number of approaches (n = 92) is not equal to the total number of studies (n = 67) because some studies applied multiple approaches

**Fig. 2:**
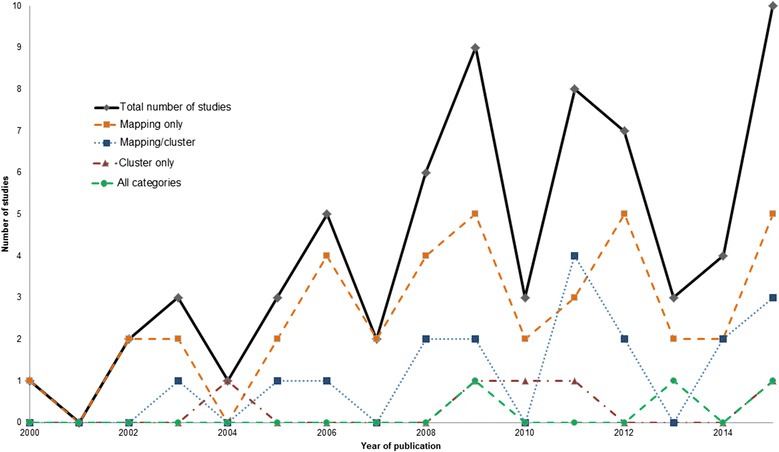
Application of geospatial analysis methods to unintentional injury data since 2000 (*n =* 67 studies)

The year of publication for the included studies, overall and by combination of categories, is presented in Fig. 2. There was an overall trend towards increased use of geospatial methods, especially clustering, since 2008, demonstrated by the increasing number of studies that applied both mapping and clustering/cluster detection methods.

### Mapping studies

Of the 62 studies identified as using mapping (Table 2), the injury cause categories most frequently investigated were road crashes (*n =* 33), falls (*n =* 10), burns (*n =* 9), drowning (*n =* 4), occupational (*n =* 2), aviation-related (*n =* 2), dog-bite (*n =* 1) and natural disaster (*n =* 1). Of the mapping studies, 15 studies presented dot maps of specific injury locations, 50 studies presented summary measures of aggregated data in choropleth (*n =* 47) and classed symbol (*n =* 3) maps. Three of the included studies presented two types of maps (dot and choropleth) so the sum of this group is not equal to the total number of studies (*n =* 65 types of maps, *n =* 62 studies). The choropleth and classed symbol maps represented different types of summary measures: incidence rate (*n =* 27), relative risk (*n =* 10), frequency (*n =* 8), and standardised mortality ratios (*n =* 6). One study mapped more than one summary measure, namely, incidence rate and relative risk (Williams et al. 2003), so again, the sum by summary measures (*n =* 48) does not equal the total number of studies (*n =* 47) presented choropleth maps.

**Table 2:** Number of studies presenting injury maps and the type of measure represented (*n =* 62 studies)

	Type of map		
	Dot	Choropleth	Classed symbol
Injury cause categories			
Road traffic (*n =* 33)	10	24	1
Falls (*n =* 10)	3	6	1
Burns (*n =* 9)	-	8	1
Drowning (*n =* 4)	1	3	-
Occupational (*n =* 2)	-	2	-
Aviation-related (*n =* 2)	-	2	-
Natural disasters (*n =* 1)	1	1	-
Dog-bite (*n =* 1)	-	1	-
Total number of studies^a^	15	47	3
Summary measures			
Incidence rates	-	27	-
Relative risk	-	10	-
Standardised mortality ratio	-	6	-
Frequency or count	15	5	3
Total number of studies^b^	15	48	3

^a^Some studies reported more than one type of map, so the sum is not equal to n = 62. ^b^One study reported choropleth maps with two summary measures, so the sum is not equal to n = 47

Table 2 summarises the types of maps and summary measures within the included studies. Most studies presented multiple maps as figures within the manuscript, representing the different variables under investigation. In thirteen studies, different smoothing techniques were applied to address small number problem. Methods used were an empirical Bayes model (*n =* 5) (de Pina et al. 2008; Erdogan 2009; Lassarre and Thomas 2005; Silva et al. 2011; Yiannakoulias et al. 2003), Bayesian model (*n =* 4) (Eksler and Lassarre 2008; Eksler et al. 2008; Turner et al. 2009; Williams et al. 2003), BYM (*n =* 3) (DiMaggio 2015; Heng et al. 2015; Poulos et al. 2012) and Poisson regression model (*n =* 1) (Spoerri et al. 2011). The most commonly used empirical Bayes method (Clayton and Kaldor 1987) determines the extent of smoothing from the underlying structure of data including the crude standardised mortality ratio, its precision and the underlying relative risk distribution. In contrast, the BYM approach (Besag et al. 1991) takes into account both spatial effects (spatial dependency) and heterogeneous effects (spatial independence) to estimate smoothed rates.

### Clustering/cluster detection studies

Table 3 summarises the characteristics of the clustering (global) or cluster detection (local) methods that were applied in 27 studies. Overall, the injury cause categories investigated were road traffic accidents (*n =* 15), falls (*n =* 6), burns (*n =* 2), drowning (*n =* 2), occupational (*n =* 1) and poisoning (*n =* 1). In total, eight different clustering/cluster detection methods were used, with 13 studies using >1 method. Four methods (NNI, NnH, Moran’s *I,* Geary’s *c*) were applied to test for clustering and four methods (Kernel Density Estimation (KDE), spatial scan statistics, LISA and Getis Ord statistics) were applied to identify clusters or hotspots.

**Table 3:** Applied cluster detection methods according to spatial resolution and global/local estimation (*n =* 27 studies)

Method	Spatial resolution	Global/ local	Total studies^a^	Injury category (number of studies)	References
Kernel density estimation	point	local	10	Road traffic (*n =* 7) Falls (*n =* 2) Drowning(*n =* 1)	(Cinnamon et al. 2011; Dai et al. 2013; Lai et al. 2009b; Lai et al. 2011; Lawrence et al. 2015; Morency and Cloutier 2006; Nagata et al. 2011; Schuurman et al. 2009; Slaughter et al. 2014; Weiner and Tepas 2009)
Nearest neighbour hierarchical	point	global	4	Falls (*n =* 3) Drowning (*n =* 1)	(Lai et al. 2009a; Lai et al. 2009b; Lai et al. 2011; Shenoi et al. 2015)
Nearest neighbour index	point	global	1	Road traffic (*n =* 1)	(Nunn and Newby 2015)
Spatial scan statistics	point or areal	local	4	Falls (*n =* 2) Occupational (*n =* 1) Poisoning(*n =* 1)	(Dey et al. 2010; Forst et al. 2015; Nkhoma et al. 2004; Yiannakoulias et al. 2003)
Moran’s *I*	areal	global	13	Road traffic (*n =* 8) Falls (*n =* 1) Burns (*n =* 2) Drowning (*n =* 1) Occupational (*n =* 1)	(de Pina et al. 2008; Erdogan 2009; Forst et al. 2015; Goltsman et al. 2014; Heng et al. 2015; Jones et al. 2008; Lassarre and Thomas 2005; Lawrence et al. 2015; Nunes and Nascimento 2012; Nunn and Newby 2015; Poulos et al. 2012; Shenoi et al. 2015; Silva et al. 2011)
Geary’s *c*	areal	global	2	Road traffic (*n =* 2)	(Erdogan 2009; Lassarre and Thomas 2005)
Local indicators of spatial association	areal	local	5	Road traffic (*n =* 3) Drowning (*n =* 1) Falls (*n =* 1)	(Dai et al. 2013; de Pina et al. 2008; Erdogan 2009; Nunes and Nascimento 2012; Nunn and Newby 2015)
Getis Ord statistics	areal	local	4	Road traffic (*n =* 3) Burn (*n =* 1)	(Erdogan 2009; Goltsman et al. 2014; Slaughter et al. 2014; Statter et al. 2011)

^a^total number of studies by injury category is not equal to (n = 27) because some studies applied more than one method in a single study

The most frequently used hotspot analysis method for point data (*n =* 10 studies) was KDE (considered to be a cluster detection method because of its ability to provide evidence of hotspots) which is mostly used for exploratory analysis of hotspots through a density map. The strength of KDE is that it provides evidence of hotspots in the visual form but the results of KDE methods are largely dependent on the bandwidth (search radius) parameter settings. (Fritz et al. 2013) This method was most commonly used in road traffic injuries (*n =* 7) followed by falls (*n =* 2) and drowning (*n =* 1). A commonly used clustering method for point data was NnH (*n =* 4), which determines clusters as standard deviational ellipses based on model parameters such as the specified threshold distance and minimum number of points to be included.

The most commonly applied method for aggregated data to test for spatial autocorrelation (*n =* 13 studies) was Moran’s *I* (Moran 1950) for which a value >1 indicates presence of spatial autocorrelation. Widely applied hotspot analysis methods for aggregated data, namely the LISA and Getis Ord statistics, were applied in four and five studies respectively (Chaney and Rojas-Guyler 2016; Jerrett et al. 2010). Spatial scan statistics, the most common method in broader epidemiological studies (Auchincloss et al. 2012), was applied in four studies with rarer injury events such as poisoning, occupational or work-related injuries. The strength of spatial scan statistics includes their ability to adjust for confounding variables, population densities and more importantly multiple testing (Auchincloss et al. 2012; Kulldorff 1997).

### Ecological studies

Three studies applied spatial regression methods to address aetiological questions. Spatial autoregressive models based on CAR (*n =* 1) and GWR (*n =* 2) frequentist approaches were applied to investigate social and environmental factors associated with road traffic mortality (Erdogan 2009) and drownings (Dai et al. 2013; Shenoi et al. 2015). One of the drowning studies (Shenoi et al. 2015) applied a CAR spatial regression model to estimate the influence of sociodemographic and environmental variables (e.g. ethnicity, number of pools by single family and multi-family buildings) on the number of childhood swimming pool submersions. Similarly, GWR was applied in another study (Dai et al. 2013) to investigate the influence of social and physical characteristics (e.g. housing density, number of pools, open water bodies, median income) and drowning densities. The road traffic mortality study (Erdogan 2009) applied GWR to investigate relationships between neighbourhood characteristics (e.g. length of roads, number of different types of vehicles) and death rates. The common rationale behind the use of spatial regression methods is to minimise the effect of spatial autocorrelation, as was illustrated by the included studies. A particular advantage of the GWR approach is that it is a local regression technique that allows aetiological relationships to vary from location to location, making it easier to interpret the results (Brunsdon et al. 1998).

## Discussion

Geospatial methods are valuable for understanding injury outcomes because they can be used to recognise patterns of occurrence, identify priority areas for prevention measures and provide more accurate modelling of clustered data that is inherently correlated (Cromley and McLafferty 2011; Ostfeld et al. 2005). While the benefits of geospatial methods have been widely known in broader public health applications for disease surveillance and data exploration in a spatial context (Auchincloss et al. 2012; Martinez et al. 2016; Rezaeian et al. 2007), this review shows that their use in the context of investigating unintentional injuries has been far less common.

Road traffic injuries were the most common category of injury causes investigated through geospatial methods. A possible reason for this could be the long-standing and well-managed injury surveillance systems for road traffic injuries that routinely collect data on the precise location of injury (e.g. specific road intersections). In addition, because there is a well-recognised and significant public health burden from these injuries, especially for fatal cases, they have long been a high priority for injury data systems development and prevention (Ameratunga et al. 2006). Outside of road traffic injuries, the use of geospatial methods has been more limited, mainly used in research of falls, burns and drowning injuries. This might be because of low counts of these injuries in a spatial context. There were some injury cause categories that were notable for their absence in the published spatial epidemiology injury studies, including injuries associated with sport and recreation, an area that could be expanded through future research.

Over the past 15 years, there appears to have been an increasing application of geospatial methods for investigating unintentional injuries, demonstrated by the growing number of published studies using these methods, particularly since 2008. This is likely due to recent advancements in geospatial methods and the development of GIS, which has now made it possible to capture, store, manipulate, analyse, manage and present all types of spatial or geographical data (Fotheringham and Rogerson 2013). It may also reflect the increased availability of routinely collected injury and determinant data that includes a spatial reference, as is now common from government and private organisations.

This review has demonstrated that mapping has been by far the most common spatial analysis approach adopted in injury epidemiological studies. Maps offer the advantage of presenting a clear visual representation of data showing regional or spatial variation in burden or injury risk (Martinez et al. 2016). Maps of standardised mortality ratios, relative risks or other similar statistical measures presented in the reviewed literature are useful for describing the spatial pattern of injury risk. However, basic mapping approaches may misrepresent spatial patterns because estimated standardised mortality ratios or other similar statistical measures do not take into account varying population sizes resulting in apparently large standardised mortality ratios in areas with small populations (Clayton and Kaldor 1987; Lawson et al. 2000). To some extent, this problem can be addressed by applying smoothing models to the risk estimates that take the overall distribution of rates into account (Rezaeian et al. 2007). Widely accepted models such as the empirical Bayes (Clayton and Kaldor 1987) and BYM (Besag et al. 1991) methods, were applied in very few of the included studies that involved small geographic areas with few cases (de Pina et al. 2008; DiMaggio 2015; Heng et al. 2015; Lassarre and Thomas 2005; Silva et al. 2011; Yiannakoulias et al. 2003).

It is fundamentally important that injury epidemiological studies begin to define spatial patterns statistically to determine whether observed clustering patterns occur by chance, or if there are statistically significant clusters that require further investigation (Pfeiffer et al. 2008). Many clustering/cluster detection methods have been developed over the past two decades based on different statistical approaches such as distance based, nearest neighbour, and scanning local rates for point and aggregated data (Auchincloss et al. 2012; Fritz et al. 2013). Our review identified that, in the context of unintentional injury research, very few clustering methods have been applied. Nonetheless, it is evident that the application of these methods has increased over the last eight years, mostly for road traffic injuries, but also falls and drowning. The statistical method regarded as having the best statistical power Tango’s maximized excess events tests (Pfeiffer et al. 2008) has yet to be applied in the context of unintentional injuries. Compared to their application in broader public health studies, other methods such as K-functions and spatial scan statistics were also not common in injury studies.

There were differences apparent in the choice of geospatial methods for clustering/cluster detection in unintentional injury studies when compared to broader public health research, suggesting that unintentional injuries might be different in terms of their spatial contexts and, hence, need to be treated differently. It is beyond the scope of this particular review to assess this more formally, but it is certainly worthy of future research attention. There are no established guidelines to suggest which method is most appropriate for what type of injury data. Largely, it appears the choice of method is dependent on what has been readily integrated into common GIS packages. Each clustering/cluster detection method will produce a different result for the same dataset and that result will also vary based on parameter settings (Fritz et al. 2013). This means that identifying the appropriate method along with parameter settings for a particular dataset is challenging and requires multiple testing. Further research in this area would be a valuable contribution.

Health outcome data routinely collected by private and government agencies is often only available as aggregated summaries for well-defined geographic areas. In such cases, spatial inferences can be made at the aggregated level in relation to socio-economic and environmental risk factors for clues to aetiology (Beale et al. 2008). The increasing availability of routinely collected injury data in the form of aggregated summaries lends itself to potential opportunities for ecological studies (Beale et al. 2008). Statistical challenges for this type of analysis include taking into account variability and potential error in rates, due to unequal population distributions and spatial autocorrelation (Elliot et al. 2000). The included studies that applied spatial regression techniques demonstrated how these methods can help to address statistical challenges associated with aggregated data by geographical regions (Dai et al. 2013; Erdogan 2009; Shenoi et al. 2015). These studies also analysed a diverse range of factors (e.g. neighbourhood, environmental characteristics) which may not be possible to assess at an individual level.

Geospatial methods play an important role in understanding the influence of complex social environments on injury outcomes that will help to develop population level injury prevention strategies (Bell and Schuurman 2010). In addition, they can help to identify which populations/sub-groups are consistently at greater (or lower) risk to inform the targeting of prevention efforts in those areas. This review has demonstrated that there is a move towards the use of more sophisticated geospatial methods from more traditional perspectives with the increasing availability in health and determinant data and also advances in GIS and other technologies. Continued advancement in this area would be well served by a detailed review of the quality of the geospatial methods currently adopted in injury epidemiological studies.

## Limitations of this review

A large number of the considered studies in the initial data selection phase investigated crash, collision or accident data without referring specifically to any injury incidence data. Some of these studies also appeared to have used the terms crash/collision/accident and injury interchangeably. This made it challenging to identify the studies that investigated injury data specifically. To address this, decisions to exclude a study were made only after agreement by two authors to help reduce the potential of excluding a publication in error.

In the reviewed literature, different terms were used to describe the application of geospatial methods in epidemiological studies (e.g. spatial epidemiology, spatial analysis, geographical variation, mapping, and geographical epidemiology). There is a possibility that some relevant keywords (eg. space-time) were missed in the search strategy because of the multidisciplinary nature of this area and the use of many colloquial words by those who work in the area. Moreover, it is possible that searching of other databases, such as the transport research international documentation, may have identified some additional relevant papers. However, given the extensive study selection process the studies identified are likely to be a highly representative sample of papers published in this area. If papers were missed, they are most likely from the category 1 studies (i.e. mapping of descriptive data), with no clear methodology indicating application of spatial methods. There is less likelihood that a study from category 2 or 3 (cluster or ecological methods) will have been missed, as authors of those studies would likely use the more familiar terminology in formal publications. Therefore, the major findings are unlikely to be influenced by any missed publications.

It should be noted that although we have categorised the studies into three distinct categories of spatial epidemiological approaches, this was to simplify the presentation of these results and understanding by a non-technical audience. In reality, these categories occur more along a continuous process rather than as discrete steps (Colantonio et al. 2011; Elliott and Wartenberg 2004; Lawson et al. 2016). Many studies used multiple categories and methods and the boundaries between them were not always clear. For example, the most comprehensive studies began by mapping raw data, further explored the data using one or more cluster detection methods and then applied one or more spatial regression methods to understand the relationship with predictor variables (Dai et al. 2013; Shenoi et al. 2015).

The aim of this review has been to provide an overview of the types of geospatial methods applied to unintentional injury epidemiological studies. This study does not provide detail of the analytical processes or steps involved in cluster detection or the spatial regression methods identified. The interested reader is advised to consult key references for specific methods that have been presented throughout the paper (including (Anselin 1995; Brunsdon et al. 1998; Fritz et al. 2013; Getis and Ord 1992; Kulldorff 1997; Marshall 1991).

## Conclusions

This review has demonstrated that the application of geospatial methods to investigations of unintentional injuries has increased over recent years, but is still relatively uncommon. The majority of studies applying geospatial methods have focused on road traffic injuries. However, other injury cause categories, particularly falls and burns, have also started to make use of geospatial methods in recent years. Mapping was the most commonly used approach for visual display of injury incidence rates. Where applied, cluster detection methods have identified statistically significant spatial dependency within the injury data under investigation. In such cases, the use of spatial regression techniques are needed to minimise the effect of spatial autocorrelation. Geospatial methods are rapidly emerging as an accessible tool for injury researchers to better understand complex injury aetiology but to date, few authors have made use of their full potential in the major injury cause categories.

## Additional files

Additional file 1: Statement of Preferred Reporting Items for Systematic Reviews and Meta-Analyses (PRISMA Statement). (DOC 63 kb)
Additional file 2: Search strategy, databases and eligibility criteria. (DOCX 15 kb)
Additional file 3: Characteristics of included studies. (DOCX 107 kb)

## Abbreviations

BYM: Besag York and Molliè
CAR: Conditional Autoregressive Models
GIS: Geographic Information System
GWR: Geographically Weighted Regression
KDE: Kernel Density Estimation
LISA: Local Indictor of Spatial Autocorrelation
NnH: Nearest neighbour Hierarchical
NNI: Nearest Neighbour Index
PRISMA: Preferred Reporting Items for Systematic Reviews and Meta-Analyses
