# Supplementary material for: An overview of geospatial methods used in unintentional injury epidemiology
Source: Inj Epidemiol. 2016 Dec 26;3:32. doi: 10.1186/s40621-016-0097-0 (PMC5183571; doi:10.1186/s40621-016-0097-0)
Supplement: Additional file 2: — Search strategy, databases and eligibility criteria. (DOCX 15 kb) [file 40621_2016_97_MOESM2_ESM.docx]

**Supplementary file 2 – Search strategy for inclusion of papers to review**

**Search terms**

#1 - Geographic variation, geographical variation, geographic distribution, geographical distribution, geographic analysis, geographic analyses, spatial clustering, spatial cluster, spatial interaction, spatial autocorrelation, spatial auto-correlation, geographical mapping, spatial analysis, spatial analyses, spatial heterogeneity, geographically weighted, hotspot, hot-spot, high risk, hotspots, hot-spots, geographic information systems, geographic information system, GIS, spatial error modelling, geospatial, geo-spatial, exploratory data analysis, spatial correlation, spatial Bayesian modelling, geographical risk factors, spatial externality, geographical characteristics, conditional autoregressive, geographical inequalities, spatial aggregation, spatiotemporal, spatio-temporal, spatial temporal, neighbourhood, spatial structure

#2 - Trauma, traumas, traumatic, injury, injuries, injured, drown, drowning, drowned, burn, burns, fall, falls, crash, crashes, accident, accidents

#3 - Violent, violence, war, suicides, gene, genes, genital, genetic, rat, cell, soil, DNA, cancer, biological, gait, animal

**Search strategy**

(#1 AND #2) NOT #3

**Databases**

Medline, Academic Search Complete, CINAHL Complete, Engineering Source, GeoRef, Health Source: Nursing/Academic Edition, PsycINFO, SPORT Discus with Full Text, Web of Science

**Eligibility criteria**

Inclusion criteria

- Unintentional injuries
- Spatial injury epidemiological studies
- Population level studies
- 2000-2015 inclusive
- Only original data studies were included

Exclusion criteria

- Crash, collision and accident data
- Suicides or social harm related deaths or injuries
