# Supplementary material for: An overview of geospatial methods used in unintentional injury epidemiology
Source: Inj Epidemiol. 2016 Dec 26;3:32. doi: 10.1186/s40621-016-0097-0 (PMC5183571; doi:10.1186/s40621-016-0097-0)
Supplement: Additional file 3: — Characteristics of included studies. (DOCX 107 kb) [file 40621_2016_97_MOESM3_ESM.docx]

**Supplementary file 3: Characteristics of included studies**

| Reference | Study area/  injury data source (year) | Injury cause |  | | | | | GIS packages | Smoothing |
| --- | --- | --- | --- | --- | --- | --- | --- | --- | --- |
|  |  |  | **Mapped variables** | **Map type** | **Clustering** | **Cluster detection** | **Geographical correlation methods** |  |  |
| ([Towne et al. 2015](#_ENREF_61)) | **Texas**  Hospital Inpatient Discharge Public UseData File  (2007- 2011) | F | IR | CM | - | - | - | ArcGIS | - |
| ([Stylianou et al. 2015](#_ENREF_59)) | **England and Wales**  international Burn Injury Database (iBID)  (2003-2011) | B | IR | CM | - | - | - | STATA | - |
| ([Shenoi et al. 2015](#_ENREF_54)) | **Harris County, Texas**  Houston Fire Department and county fatality records  (2003-2007) | D | FM | DM | NnH,  Moran's I | - | CAR | CrimeStat, ArcGIS | - |
| ([Nunn and Newby *2*015](#_ENREF_46)) | **Indiana**  Indiana State Police Automated Reporting Information Exchange System (ARIES)  (2003-2011) | R | FM,IR | DM/CM | NNI,  Moran's I | LISA | - | STATA, ArcGIS | - |
| ([Mohan et al. 2015](#_ENREF_39)) | **Vellore, India**  District Police Superintendent’s office  (Jan 2005-May 2007) | R | FM | CSM | - | - | - | ArcGIS | - |
| ([Lawrence et al. 2015](#_ENREF_36)) | **Melbourne**  State of Victoria’s road authority (VicRoads)  (2000-2011) | R | - | - | Moran's I | KDE | - | ArcGIS | - |
| ([Bamzar and Ceccato 2015](#_ENREF_1)) | **Sweden**  Swedish National Board of Health and Welfare  (2001-2010) | F | IR | CM | - | - | - | - | - |
| ([Heng et al. 2015](#_ENREF_24)) | **London**  International Burn Injury Database (iBID)  (2007-2013) | B | RR | CM | Moran's I | - | - | R | BYM |
| ([Forst et al. 2015](#_ENREF_16)) | **Illinois**  State of Illinois trauma registry (ITR)  (2000-2009) | O | FM | CM | Moran's I , | SaTScan | - | SaTScan | - |
| ([DiMaggio 2015](#_ENREF_9)) | **New York**  New York City Department of Transportation  (2001-2010) | R | RR | CM | - | - | - | R | BYM |
| ([Slaughter et al. 2014](#_ENREF_56)) | **New York**  Bellevue Hospital, trauma center in New York City  (Dec 2000-Jun 2011) | R | FM | DM | - | KDE,  Getis ord statistics | - | Maptitude, ArcGIS | - |
| ([Raghavan et al. 2014](#_ENREF_50)) | **Manitoba, Canada**  Population Health Research Data Repository, Manitoba Centre for Health Policy, University of Manitoba  (1984-2006) | Dog-bite | IR | CM | - | - | - | SAS | - |
| ([Mian et al. 2014](#_ENREF_38)) | **Georgia and South Carolina**  National Trauma Registry of the American College of Surgeons data set  (2006–2009) | B | IR | CM | - | - | - | ArcGIS | - |
| ([Goltsman et al. 2014](#_ENREF_18)) | **New South Wales**  New South Wales Severe Burns Injury Service  (2006-2010) | B | RR | CM | Moran's I | Getis ord statistics | - | ArcGIS | - |
| ([Hosking et al. 2013](#_ENREF_26)) | **Auckland**  New Zealand National Minimum Data Set  (2000-2008) | R | IR | CM | - | - | - | ArcGIS | - |
| ([Harlan et al. 2013](#_ENREF_21)) | **Maricopa,** **Arizona**  Maricopa County Department of Public Health  (2000-2008) | B | FM | CSM | - | - | - | - | - |
| ([Dai et al. 2013](#_ENREF_6)) | **Georgia**  Child Advocate in Georgia  (2002-2008) | D | IR | CM | - | KDE, LISA | GWR | ArcGIS | - |
| ([Unni et al. 2012](#_ENREF_63)) | **Middle Tennessee**  Trauma registry, pediatric trauma center  (2007-2009) | R | IR | CM | - | - | - | ArcGIS | - |
| ([Sharif et al. 2012](#_ENREF_53)) | **Texas**  Storm Data publication  (1959-2009) | D | FM | CM | - | - | - | - | - |
| ([Poulos et al. 2012](#_ENREF_49)) | **New South Wales**  NSW Admitted Patients Data Collection  (2000-2005) | R | RR | CM | Moran's I | - | - | MapInfo | BYM |
| ([Morency et al. 2012](#_ENREF_41)) | **Laval and Montréal Island,** **Canada**  Ambulance services  (Dec 2008-Jan 2009) | F | FM | CSM | - | - | - | ArcGIS | - |
| ([Huff et al. 2012](#_ENREF_28)) | **Texas**  Trauma registry data  (2004-2010) | R | IR | CM | - | - | - | ArcGIS | - |
| ([Chan et al. 2012](#_ENREF_4)) | **WellingtoneDufferineGuelph health region,** **Ontario**, **Canada**  Ontario provincial health planning database  (2002-2006) | F | RR | CM | - | - | - | WinBUGS,  ArcGIS | - |
| ([Nunes and Nascimento 2012](#_ENREF_45)) | **São Paulo**, **Brazil**  Unified Health System  (2007-2009) | R | SMR | CM | Moran's I | LISA | - | Terraview | - |
| ([Statter et al. 2011](#_ENREF_58)) | **Service area of the Hospital, Chicago**  University of Chicago Medical Center pediatric trauma center  (2002-2009) | R | FM | DM | - | Getis ord statistics | - | - | - |
| ([Spoerri et al. 2011](#_ENREF_57)) | **Switzerland**  RTA mortality records  (2000–2005) | R | SMR | CM | - | - | - | WinBUGS,  STATA | Poisson regression model |
| ([Silva et al. 2011](#_ENREF_55)) | **Pernambuco**, **Northeastern Brazil**  State of Pernambuco  (2000-2005) | R | IR | CM | Moran's I | - | - | TerraView | Empirical Bayes model |
| ([Razzak et al. 2011](#_ENREF_51)) | **Karachi**  medico-legal office  (Jan 2004-Dec 2004) | R | FM | DM | - | - | - | ArcGIS | - |
| ([Lateef 2011](#_ENREF_35)) | **Karachi**  Major trauma centres  (2008) | R | FM | DM | - | - | - | - | - |
| ([Lai et al. 2011](#_ENREF_33)) | **Hongkong**  Kwong Wah Hospital  (2006–2007) | F | FM | DM | NnH | KDE | - | - | - |
| ([Nagata et al. 2011](#_ENREF_42)) | **Hanoi**  Hanoi city police agency  (Jan 2006-Dec 2006) | R | FM | DM | - | KDE | - | ArcGIS | - |
| ([Cinnamon et al. 2011](#_ENREF_5)) | **British Columbia**, **Canada**  Insurance Corporation of British Columbia’s pedestrian injury dataset  (2000-2005) | R | - | - | - | KDE |  | ArcGIS | - |
| ([Edelman et al. 2010](#_ENREF_12)) | **Utah**  State databases  (1997-2001) | B | RR | CM | - | - | - | ArcGIS | - |
| ([Dey et al. 2010](#_ENREF_8)) | **United states of America**  Centers for Disease Control and Prevention BioSense system  (Nov 2007-Mar 2008) | F | - | - | - | Spatial scan statistics | - | SaTScan | - |
| ([Chakravarthy et al. 2010](#_ENREF_3)) | **California**  California Statewide Integrated Traffic Record Systems  (2000-2004) | R | FM | DM | - | - | - | ArcGIS | - |
| ([Weiner and Tepas 2009](#_ENREF_64)) | **Jacksonville**, **Florida**  ED and trauma registry data  (2002-2006) | R | FM | DM | - | KDE |  | ArcGIS | - |
| ([Turner et al. 2009](#_ENREF_62)) | **New South Wales**  NSW Inpatient Statistics Collection  (Jul 1998-Jun 2004) | F | RR | CM | - | - | - | - | Bayesian model |
| ([Sukhai et al. 2009](#_ENREF_60)) | **South Africa**  National Department of  Transport (NDoT)  (2002-2006) | R | IR | CM | - | - | - | Arc GIS | - |
| ([Schuurman et al. 2009](#_ENREF_52)) | **British Columbia**, Canada  Insurance Corporation of British Columbia and BCTR  (2000 to 2005) | R | - | - | - | KDE | - | ArcGIS | - |
| ([Lai Martin et al. 2009](#_ENREF_32)) | **Mong Kok,** Hong Kong  Telephone interview of patients attending Kwong Wah Hospital as a result of fall, (July 2006-Sept 2006, Jan 2007-Dec 2007) | F | FM | DM | NnH | KDE | - | ArcGIS | - |
| ([Maples and Tiefenbacher 2009](#_ENREF_37)) | **Texas**  National Climatic Data Center Storm Events database, Spatial Hazard Events and Losses Database for the United States database (1950-2004) | D | FM | CM | - | - | - | - | - |
| ([Lai Low et al. 2009](#_ENREF_31)) | **Mong Kok, Hong Kong**  Telephone interview of patients attending Kwong Wah Hospital as a result of fall, (July 2006-Sept 2006, Jan 2007-Dec 2007) | F | FM | DM | NnH | - | - | CrimeStat | - |
| ([Erdogan 2009](#_ENREF_15)) | **Turkey**  Police and gendarmerie reports  (2001-2006) | R | IR | CM | Moran's I, | Garey c, LISA,  Getis ord statistics | GWR | ArcGIS, GeoDa, CrimeStat | Empirical Bayes model |
| ([Dissanayake et al. 2009](#_ENREF_10)) | **Newcastle**, **England**  Tyne and Wear Traffic Accident and Data Unit  (2000-2005) | R | FM | DM | - | - | - | ArcGIS | - |
| ([Jones et al. 2008](#_ENREF_29)) | **England and Wales**  Stats19  (1995–2000) | R | FM | CM | Moran's I | - | - | ArcGIS | - |
| ([Hu et al. 2008](#_ENREF_27)) | **China**  Transportation and communications yearbook  (1986–2006) | R | IR | CM | - | - | - | EpiInfo | - |
| ([Haynes et al. 2008](#_ENREF_23)) | **New Zealand**  Crash Analysis System based on highway -police reports and maintained by Land Transport New Zealand  (1996-2005) | R | IR | CM | - | - | - | - | - |
| ([Eksler et al. 2008](#_ENREF_14)) | **25 European Union (EU)**  National road administration and statistical offices  (2002) | R | RR | CM | - | - | - | WinBUGS | Bayesian model |
| ([Eksler and Lassarre 2008](#_ENREF_13)) | **Belgium**  Belgian National Statistical Office  (2000-2005) | R | RR | CM | - | - |  | WinBUGS | Bayesian model |
| ([de Pina et al. 2008](#_ENREF_7)) | **Portugal**  National Hospital Discharge Register  (2000-2002) | F | IR | CM | Moran's I | LISA | - | - | Empirical Bayes model |
| ([La Torre et al. 2007](#_ENREF_30)) | **Italy**  Statistics of crash accidents Year 2001  (1999-2001) | R | IR | CM | - | - | - | - | - |
| ([Breslin et al. 2007](#_ENREF_2)) | **Ontario**  Ontario  Workplace Safety and Insurance Board  (2000) | O | IR | CM | - | - | - | - | - |
| ([Yan-Hong et al. 2006](#_ENREF_66)) | **Shanghai**  Traffic Administration Bureau, Shanghai’s 494 hospitals  (1987-2003) | R | IR | CM | - | - | - | ArcGIS | - |
| ([Paulozzi 2006](#_ENREF_47)) | **United States of America**  Centers for Disease Control and Prevention's Web-based Injury Statistics Query and Reporting System  (1999-2002) | R | IR | CM | - | - | - | EpiInfo | - |
| ([Niekerk et al. 2006](#_ENREF_43)) | **Cape Town**  Red Cross Children’s Hospital register  (Jan 1999 - Dec 2000) | B | IR | CM | - | - | - | - | - |
| ([Morency and Cloutier 2006](#_ENREF_40)) | **Montreal**, Canada  Ambulance services information systems  (1999-2003) | R | FM | DM/CM | KDE | - | - | ArcGIS | - |
| ([Fouillet et al. 2006](#_ENREF_17)) | **Paris**  Centre d’Epidémiologie sur les Causes médicales de décès (Cépi-Dc) of INSERM  (2000-2003) | B | SMR | CM | - | - | - | - | - |
| ([Lassarre and Thomas 2005](#_ENREF_34)) | **Europe**  The international road traffic accident database  (1998) | R | SMR | CM | Moran's I, Garey C | - | - | - | Emprical Bayes model |
| ([Haynes et al. 2005](#_ENREF_22)) | **England and Wales**  Stats 19  (1995–1999) | R | SMR | CM | - | - | - | - | - |
| ([Durkin et al. 2005](#_ENREF_11)) | **Wisconsin**  Wisconsin Crash Outcome Data Evaluation System (CODES)  (1992-2001) | R | IR | CM | - | - | - | ArcGIS | - |
| ([Nkhoma et al. 2004](#_ENREF_44)) | **Texas**  Centers for Disease Control and Prevention  (1980-2001) | P | - | - | - | Spatial scan statistics | - | SaTScan | - |
| ([Yiannakoulias et al. 2003](#_ENREF_67)) | **Alberta**  Administrative health data systems, Alberta Health and Wellness  (1997–98) | F | IR | CM | - | Spatial scan statistics | - | SaTScan | Empirical Bayes model |
| ([Williams et al. 2003](#_ENREF_65)) | **St. Louis**  Two Children hospital  (1995) | B | IR/RR | CM | - | - | - | ArcGIS, WinBUGS | Bayesian models |
| ([Hijar et al. 2003](#_ENREF_25)) | **Mexico**  Death certificates  (1994-1997) | R | SMR | CM | - | - | - | MapInfo | - |
| ([Grabowski et al. 2002a](#_ENREF_19)) | **United States of America**,  NTSB factual reports  (1983-1998) | Av | IR | CM | - | - | - | ArcGIS | - |
| ([Grabowski et al. 2002b](#_ENREF_20)) | **United States of America**  NTSB factual reports  (1983-1998) | Av | IR | CM | - | - | - | ArcGIS | - |
| ([Peek-Asa et al. 2000](#_ENREF_48)) | **Los Angeles**  County Coroner's Office/hospitals in Los Angeles County  (1994) | ND | FM/IR | DM/CM | - | - | - | ArcGIS | - |
| F = Fall , B = Burn, R = Road traffic, D = Drowning, O = Occupational or work-related, P = Poisoning, ND = Natural disasters, Av = Aviation-related, IR = incidence rate, RR = Relative risk, SMR = Standardised mortality ratio, FM = Frequency, DM = Dot maps, CM = Choropleth Maps, CSM = Classed symbol maps, GWR = Geographically weighted regression, BYM = Besag-York-Mollié, NNI = Nearest neighbour index, NnH = Nearest neighbour hierarchical, KDE = Kernel density estimation, LISA = Local indicator of spatial autocorrelation, CAR = Conditional auto-regressive | | | | | | | | | |

**References**

Bamzar R, Ceccato V. The nature and the geography of elderly injuries in Sweden. GeoJournal. 2015;80(2):279-299.

Breslin FC, Smith P, Dunn JR. An ecological study of regional variation in work injuries among young workers. BMC Public Health. 2007; doi:10.1186/1471-2458-7-91

Chakravarthy B, Anderson CL, Ludlow J, Lotfipour S, Vaca FE. The relationship of pedestrian injuries to socioeconomic characteristics in a large southern California county. Traffic Inj Prev. 2010;11(5):508-513.

Chan WC, Law J, Seliske P. Bayesian spatial methods for small-area injury analysis: a study of geographical variation of falls in older people in the Wellington -Dufferine-Guelph health region of Ontario, Canada. Inj Prev. 2012;18(5):303-308.

Cinnamon J, Schuurman N, Hameed SM. Pedestrian injury and human behaviour: observing road-rule violations at high-incident intersections. PLoS ONE. 2011; doi:10.1371/journal.pone.0021063

Dai D, Zhang Y, Lynch CA, Miller T, Shakir M. Childhood drowning in Georgia: a geographic information system analysis. Appl Geogr. 2013;37:11-22.

de Pina MF, Alves SM, Barbosa M, Barros H. Hip fractures cluster in space: an epidemiological analysis in Portugal. Osteoporos Int. 2008;19(12):1797-1804.

Dey AN, Hicks P, Benoit S, Tokars JI. Automated monitoring of clusters of falls associated with severe winter weather using the BioSense system. Inj Prev. 2010;16(6):403-407.

DiMaggio C. Small-Area Spatiotemporal Analysis of Pedestrian and Bicyclist Injuries in New York City. Epidemiology. 2015;26(2):247-254.

Dissanayake D, Aryaija J, Wedagama DM. Modelling the effects of land use and temporal factors on child pedestrian casualties. Accid Anal Prev. 2009;41(5):1016-1024.

Durkin M, McElroy J, Guan H, Bigelow W, Brazelton T. Geographic analysis of traffic injury in Wisconsin: impact on case fatality of distance to level I/II trauma care. WMJ. 2005;104(2):26-31.

Edelman LS, Cook LJ, Saffle JR. Burn injury in Utah: demographic and geographic risks. J Burn Care Res. 2010;31(3):375-384.

Eksler V, Lassarre S. Evolution of road risk disparities at small-scale level: example of Belgium. J Safety Res. 2008;39(4):417-427.

Eksler V, Lassarre S, Thomas I. Regional analysis of road mortality in Europe. Public Health. 2008;122(9):826-837.

Erdogan S. Explorative spatial analysis of traffic accident statistics and road mortality among the provinces of Turkey. J Safety Res. 2009;40(5):341-351.

Forst L, Friedman L, Chin B, Madigan D. Spatial Clustering of Occupational Injuries in Communities. Am J Public Health. 2015; doi:10.2105/ajph.2015.302595.

Fouillet A, Rey G, Laurent F, Pavillon G, Bellec S, Ghihenneuc-Jouyaux C, et al., Excess mortality related to the August 2003 heat wave in France. Int Arch Occup Environ Health. 2006;80(1):16-24.

Goltsman D, Li Z, Bruce E, Maitz PK. Geospatial and epidemiological analysis of severe burns in New South Wales by residential postcodes. Burns. 2014;40(4):670-682.

Grabowski JG, Curriero FC, Baker SP, Li G. Exploratory spatial analysis of pilot fatality rates in general aviation crashes using geographic information systems. Am J Epidemiol. 2002a;155(5):398-305.

Grabowski JG, Curriero FC, Baker SP, Li G. Geographic patterns of pilot fatality rates in commuter and air taxi crashes. Aviat Space Environ Med. 2002b;73(10):1014-1020.

Harlan SL, Declet-Barreto JH, Stefanov WL, Petitti DB. Neighborhood effects on heat deaths: social and environmental predictors of vulnerability in Maricopa County, Arizona. Environ Health Perspect. 2013;121(2):197-204.

Haynes R, Jones A, Harvey I, Jewell T, Lea D. Geographical distribution of road traffic deaths in England and Wales: place of accident compared with place of residence. J Public Health. 2005;27(1):107-111.

Haynes R, Lake IR, Kingham S, Sabel CE, Pearce J, Barnett R. The influence of road curvature on fatal crashes in New Zealand. Accid Anal Prev. 2008;40(3):843-850.

Heng JS, Atkins J, Clancy O, Takata M, Dunn KW, Jones I, et al., Geographical analysis of socioeconomic factors in risk of domestic burn injury in London 2007-2013. Burns. 2015;41(3):437-445.

Hijar M, Trostle J, Bronfman M. Pedestrian injuries in Mexico: a multi-method approach. Soc Sci Med. 2003;57(11):2149-2159.

Hosking J, Ameratunga S, Exeter D, Stewart J, Bell A. Ethnic, socioeconomic and geographical inequalities in road traffic injury rates in the Auckland region. Aust N Z J Public Health. 2013;37(2):162-167.

Hu G, Wen M, Baker TD, Baker SP. Road-traffic deaths in China, 1985-2005: threat and opportunity. Inj Prev. 2008;14(3):149-153.

Huff SD, McGaha PK, Reed M, Kratz M, Peters JA, Atkinson V. All-terrain vehicle injuries in Texas, mapping the path to intervention with a geographic information system. J Agromedicine. 2012;17(1):51-62.

Jones AP, Haynes R, Kennedy V, Harvey IM, Jewell T, Lea D. Geographical variations in mortality and morbidity from road traffic accidents in England and Wales. Health Place. 2008;14(3):519-535.

La Torre G, Van Beeck E, Quaranta G, Mannocci A, Ricciardi W. Determinants of within-country variation in traffic accident mortality in Italy: a geographical analysis. Int J Health Geogr. 2007; doi:10.1186/1476-072x-6-49

Lai PC, Low CT, Wong M, Wong WC, Chan MH. Spatial analysis of falls in an urban community of Hong Kong. Int J Health Geogr. 2009;8:14.

Lai PC, Martin W, Ming-Houng C, Wing-Cheung W, Chien-Tat L. An ecological study of physical environmental risk factors for elderly falls in an urban setting of Hong Kong. Sci Total Environ. 2009;407(24):6157-6165.

Lai PC, Wong WC, Low CT, Wong M, Chan MH. A small-area study of environmental risk assessment of outdoor falls. J Med Syst. 2011;35(6):1543-1552.

Lassarre S, Thomas I. Exploring road mortality ratios in Europe: National versus regional realities. J R Stat Soc Ser A Stat Soc. 2005;168(1):127-144.

Lateef MU. Spatial patterns monitoring of road traffic injuries in Karachi metropolis. Int J Inj Contr Saf Promot. 2011;18(2):97-105.

Lawrence BM, Stevenson MR, Oxley JA, Logan DB. Geospatial analysis of cyclist injury trends: an investigation in Melbourne, Australia. Traffic Inj Prev. 2015;16(5):513-518.

Maples LZ, Tiefenbacher JP. Landscape, development, technology and drivers: The geography of drownings associated with automobiles in Texas floods, 1950-2004. Appl Geogr. 2009;29(2):224-234.

Mian MA, Haque A, Mullins RF, Fiebiger B, Hassan Z. Urban-rural dichotomy of burn patients in Georgia and South Carolina: a geographic information system study. J Burn Care Res. 2014; doi:10.1097/bcr.0000000000000025

Mohan VR, Sarkar R, Abraham VJ, Balraj V, Naumova EN. Differential patterns, trends and hotspots of road traffic injuries on different road networks in Vellore district, southern India. Trop Med Int Health. 2015;20(3):293-303.

Morency P, Cloutier MS. From targeted "black spots" to area-wide pedestrian safety. Inj Prev. 2006;12(6):360-364.

Morency P, Voyer C, Burrows S, Goudreau S. Outdoor falls in an urban context: winter weather impacts and geographical variations. Can J Public Health. 2012;103(3):218-222.

Nagata T, Takamori A, Kimura Y, Kimura A, Hashizume M, Nakahara S. Trauma center accessibility for road traffic injuries in Hanoi, Vietnam. J Trauma Manag Outcomes. 2011;5:11.

Niekerk AV, Reimers A, Laflamme L. Area characteristics and determinants of hospitalised childhood burn injury: a study in the city of Cape Town. Public Health. 2006;120(2):115-124.

Nkhoma ET, Ed Hsu C, Hunt VI, Harris AM. Detecting spatiotemporal clusters of accidental poisoning mortality among Texas counties, U.S., 1980 - 2001. Int J Health Geogr. 2004;3(1):25.

Nunes MN, Nascimento LF. Spatial analysis of deaths due to traffic accidents, before and after the Brazilian Drinking and Driving Law, in micro-regions of the state of Sao Paulo, Brazil. Rev Assoc Med Bras. 2012;58(6):685-690.

Nunn S, Newby W. Landscapes of Risk: The Geography of Fatal Traffic Collisions in Indiana, 2003 to 2011. Prof Geogr. 2015;67(2):269-281.

Paulozzi LJ. Is it safe to walk in the Sunbelt? Geographic variation among pedestrian fatalities in the United States, 1999-2003. J Safety Res. 2006;37(5):453-459.

Peek-Asa C, Ramirez MR, Shoaf K, Seligson H, Kraus JF. GIS mapping of earthquake-related deaths and hospital admissions from the 1994 Northridge, California, Earthquake. Ann Epidemiol. 2000;10(1):5-13.

Poulos RG, Chong SS, Olivier J, Jalaludin B. Geospatial analyses to prioritize public health interventions: a case study of pedestrian and pedal cycle injuries in New South Wales, Australia. Int J Public Health. 2012;57(3):467-475.

Raghavan M, Martens PJ, Burchill C. Exploring the relationship between socioeconomic status and dog-bite injuries through spatial analysis. Rural Remote Health. 2014;14(3):2846.

Razzak JA, Khan UR, Jalal S. Application of geographical information system (GIS) for mapping road traffic injuries using existing source of data in Karachi, Pakistan--a pilot study. J Pak Med Assoc. 2011;61(7):640-643.

Schuurman N, Cinnamon J, Crooks VA, Hameed SM. Pedestrian injury and the built environment: an environmental scan of hotspots. BMC Public Health. 2009;9:233.

Sharif HO, Hossain MM, Jackson T, Bin-Shafique S. Person-place-time analysis of vehicle fatalities caused by flash floods in Texas. Geomatics Natural Hazards & Risk. 2012;3(4):311-323.

Shenoi RP, Levine N, Jones JL, Frost MH, Koerner CE, Fraser JJ, Jr., Spatial analysis of paediatric swimming pool submersions by housing type. Inj Prev. 2015;21(4):245-253.

Silva PH, Lima ML, Moreira Rda S, Souza WV, Cabral AP. Spatial study of mortality in motorcycle accidents in the State of Pernambuco, Northeastern Brazil. Rev Saude Publica. 2011;45(2):409-415.

Slaughter DR, Williams N, Wall SP, Glass NE, Simon R, Todd SR, et al., A community traffic safety analysis of pedestrian and bicyclist injuries based on the catchment area of a trauma center. J Trauma Acute Care Surg. 2014;76(4):1103-1110.

Spoerri A, Egger M, von Elm E. Mortality from road traffic accidents in Switzerland: longitudinal and spatial analyses. Accid Anal Prev. 2011;43(1):40-48.

Statter M, Schuble T, Harris-Rosado M, Liu D, Quinlan K. Targeting pediatric pedestrian injury prevention efforts: teasing the information through spatial analysis. J Trauma. 2011;71 Suppl 2:511-516.

Stylianou N, Buchan I, Dunn KW. A review of the international Burn Injury Database (iBID) for England and Wales: descriptive analysis of burn injuries 2003-2011. BMJ Open. 2015; doi:10.1136/bmjopen-2014-006184.

Sukhai A, Jones AP, Haynes R. Epidemiology and risk of road traffic mortality in South Africa. S Afr Geogr J. 2009;91(1):4-15.

Towne SD, Jr., Smith ML, Yoshikawa A, Ory MG. Geospatial distribution of fall-related hospitalization incidence in Texas. J Safety Res. 2015;53:11-16.

Turner RM, Hayen A, Dunsmuir WT, Finch CF. Spatial temporal modeling of hospitalizations for fall-related hip fractures in older people. Osteoporos Int. 2009;20(9):1479-1485.

Unni P, Morrow SE, B LS. Analysis of pediatric all-terrain vehicle trauma data in Middle Tennessee: implications for injury prevention. J Trauma Acute Care Surg. 2012;73 Suppl 3:277-280.

Weiner EJ, Tepas JJ, 3rd. Application of electronic surveillance and global information system mapping to track the epidemiology of pediatric pedestrian injury. J Trauma. 2009;66 Suppl 3:S10-16.

Williams KG, Schootman M, Quayle KS, Struthers J, Jaffe DM. Geographic variation of pediatric burn injuries in a metropolitan area. Acad Emerg Med. 2003;10(7):743-752.

Yan-Hong L, Rahim Y, Wei L, Gui-Xiang S, Yan Y, De Ding Z, et al., Pattern of traffic injuries in Shanghai: implications for control. Int J Inj Contr Saf Promot. 2006;13(4):217-225.

Yiannakoulias N, Rowe BH, Svenson LW, Schopflocher DP, Kelly K, Voaklander DC. Zones of prevention: the geography of fall injuries in the elderly. Soc Sci Med. 2003;57(11):2065-2073.
